# The Effects of Solvation Enthalpy, Surface Tension, and Conductivity of Common Additives on Positive Electrospray Ionization in Selected Pharmaceuticals

**DOI:** 10.3390/molecules30091885

**Published:** 2025-04-23

**Authors:** Pieter Venter

**Affiliations:** Biomedical Research and Innovation Platform, South African Medical Research Council, P.O. Box 19070, Tygerberg, Cape Town 7505, South Africa; pieter.venter@mrc.ac.za

**Keywords:** positive electrospray ionization, mobile phase additives, hydration enthalpy, surface tension, electrical conductivity

## Abstract

This study investigates the effects of common additives, which provide distinct proton sources—ammonium (NH_4_^+^) and hydronium (H_3_O^+^)—along with their corresponding conjugate base species, on signal intensity in positive ionization mode. The findings reveal that signal intensity is influenced by factors such as solvation enthalpy, surface tension, and conductivity. At lower additive concentrations (<10 mM), based on fold changes, no clear distinction could be made between formic acid, acetic acid, and their corresponding salts. At higher additive concentrations, NH_4_^+^ appears to be a more efficient proton source than H^+^ (H_3_O^+^), likely due to its more positive solvation enthalpy, which promotes greater enrichment of NH_4_^+^ on the droplet surface, as well as the reduced surface tension of ammonium salts compared to their acid counterparts. Additionally, ammonium hydroxide proves to be the most effective ammonium-based modifier, likely due to its anionic conjugate base, hydroxide, which has a more negative solvation enthalpy compared to acetate and formate. This characteristic is hypothesized to reduce charge neutralization of cations on the droplet surface and/or in the gas phase. Furthermore, ammonium hydroxide exhibits lower conductivity compared to the other ammonium additives, which is believed to enhance signal intensity. Ammonium bicarbonate, the second most effective additive, uniquely prevents metal adduct formation, leading to enhanced [M + H]^+^ ion signals.

## 1. Introduction

In electrospray ionization (ESI) mass spectrometry (MS), the addition of acidic or basic additives is a common practice to enhance detection sensitivity and improve chromatographic peak performance. Moreover, the high voltage (typically 2–5 kV) applied at the capillary tip during ESI induces electrophoretic charge separation of ions on the droplet surface, leading to the enrichment of a single ion polarity—either positive or negative—depending on the selected ionization mode [1]. In positive ion mode, the capillary tip is positively charged, leading to an enrichment of positive ions at the surface of the resulting droplets while the droplet interior contains uncharged molecules and neutral salts [2]. When an ammonium-based additive is used, the excess charge on the droplet surface is predominantly composed of NH_4_^+^ ions. These ions play a key role in ionizing neutral molecules, either at the droplet surface or in the gas phase.

Given this, while it is standard practice to compare different additives to enhance ionization intensity in electrospray ionization, there has been little discussion on the key factors of what makes an efficient proton source. For instance, in small molecule analysis, which proton source—NH_4_^+^ or H^+^—is more effective when the conjugate base remains constant, and what underlying factors contribute to its performance? Likewise, when using a fixed proton source such as NH_4_^+^, how do different conjugate bases—formate, acetate, bicarbonate, and hydroxide—influence signal intensity, and what drives these variations?

Research that has focused on the impact of conjugate base species on signal intensity while the proton source stays constant includes a study by Mirza and Chait [3]. In particular, Mirza and Chait [3] found that anions ranked CCl_3_COO^−^ > CF_3_COO^−^ > CH_3_COO^−^ ≈ Cl^−^ reduced the ion signal intensity and charge state of bovine cytochrome C and a short peptide in positive ion mode. They proposed a two-step mechanism: first, the anion associates with the positively charged protein in solution; second, during desolvation or in the gas phase, the ion pair separates, producing a neutral acid and a peptide with a lower charge state. Also, multiple studies have shown that the bicarbonate anion (using NH_4_HCO_3_) significantly enhances [M + H]⁺ signal intensity in positive ion mode for modified nucleosides [4,5] and DNA adducts [6,7,8] compared to formic acid, ammonium formate, and ammonium acetate. This effect is attributed to bicarbonate’s ability to inhibit metal adduct formation ([M + Na]⁺, [M + K]⁺). Additionally, Wang and co-workers [6] proposed that HCO_3_^−^ converts to carbonic acid, which then thermally decomposes into CO_2_ and water, a process that is expected to enhance protonation. Lastly, McIndoe and colleagues [9,10] demonstrated that the solvation of counterions (anions) plays a significant role in positive electrospray ionization. Their findings revealed that greater solvation of anions in positive ionization mode enhances the signal intensity of intrinsic positive ions. In addition to the effect of conjugate base species on signal intensity, surface tension and conductivity are key factors to consider when evaluating additives. Additives that lower surface tension generally enhance signal intensity, while those that provide moderate (but not excessive) conductivity can also improve ionization efficiency [11,12,13,14].

**Figure 1:**
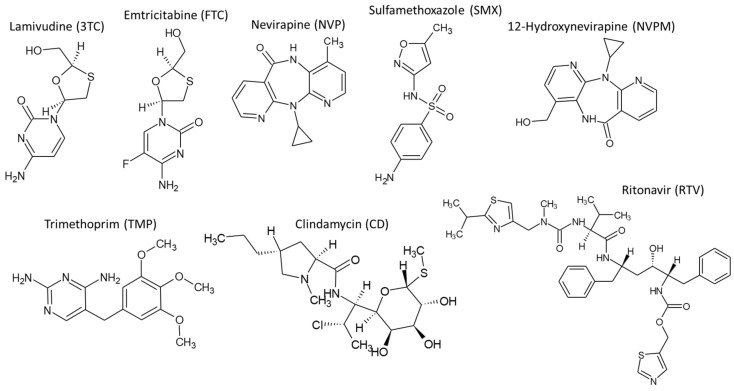
Structures of the antiretrovirals and antibiotics used in this study.

Building on these findings, the present study compares signal intensity across common mobile phase additives—ammonium acetate, ammonium formate, ammonium bicarbonate, ammonium hydroxide, formic acid, and acetic acid—by analyzing selected pharmaceutical compounds based solely on their ability to ionize more effectively in positive ionization mode compared to negative ionization mode. This would help reveal any differences in signal intensity between additives, should they exist. The compounds analyzed include antiretrovirals [15] and antibiotics (unpublished data) shown in Figure 1. Regarding the methodology, we examined different proton sources (NH_4_⁺ from ammonium acetate and formate, and H⁺ from formic and acetic acids) while keeping the conjugate base species constant (formate and acetate), and then examined the impact of various conjugate bases (formate, acetate, bicarbonate, and hydroxide) by using NH_4_⁺ as the proton source. This was achieved using a reverse-phase column coupled to a triple quadrupole mass spectrometer equipped with an electrospray ionization source operating in multiple reaction monitoring (MRM) mode.

## 2. Results and Discussion

The data shown in Figure 2 show the positive ion responses (chromatographic peak areas) for ammonium hydroxide, ammonium bicarbonate, ammonium acetate, ammonium formate, formic acid, and acetic acid across a concentration range of 1–100 mM. Representative chromatograms of all additives at concentrations ranging from 1 to 100 mM are provided for acetic acid (Figures S1–S7), formic acid (Figures S8–S14), ammonium acetate (Figures S15–S21), ammonium formate (Figures S22–S28), ammonium hydroxide (Figures S29–S35), and ammonium bicarbonate (Figures S36–S42). In general, the tendency for enhanced positive ionization follows the following approximate order:

NH_4_OH > NH_4_HCO_3_ > NH_4_CH_3_CO_2_ > NH_4_HCO_2_ > CH_3_COOH > HCOOH (Figure S43a–d).

This sequence is only a rough guideline and was determined by calculating fold changes (see Supplementary Information—Excel spreadsheet), where relevant comparisons were made, and by summing the peak areas for all analytes using a specific additive over the concentration ranges of 1–100 mM, 2.5–100 mM, 5–100 mM, and 10–100 mM, as presented in Figure S43a–d. As discussed below, the relative performance of each additive depends on concentration and can shift depending on the analyte’s chemical nature.

**Figure 2:**
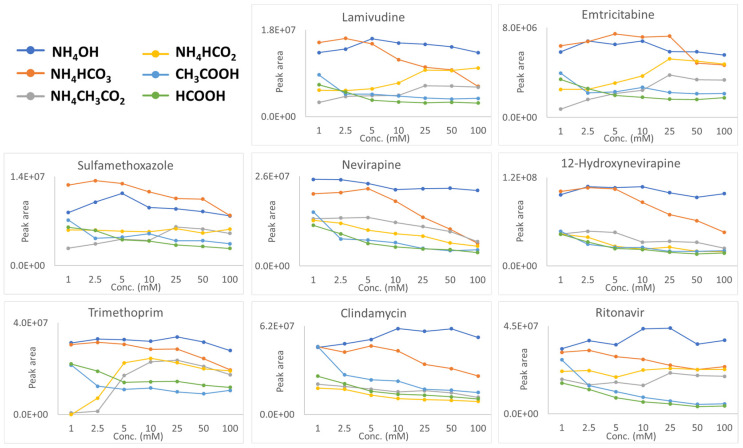
Chromatographic peak areas in positive ionization mode with various mobile phase additives across a concentration range of 1–100 mM.

To explore why, the initial comparison aimed to identify which proton source—NH_4_^+^ or H^+^—facilitated more effective positive ionization. Firstly, although ammonium hydroxide and ammonium bicarbonate are superior to formic acid and acetic acid (see Figure 2), a direct comparison cannot be made due to differences in their conjugate base species, which will be shown to play a significant role and will be discussed in the following section. Accordingly, this assessment compares formic acid and acetic acid to their corresponding salts, ammonium formate and ammonium acetate. At additive concentrations below 10 mM, no clear differences in signal intensity were observed between the acids and their corresponding salts. However, at concentrations above 10 mM, a more pronounced difference emerged, with salts generally exhibiting higher signal intensities than their acidic counterparts. This was determined by comparing the average fold change in signal intensity between two concentration ranges: 1–5 mM and 10–100 mM.

The results showed that ammonium acetate, compared to acetic acid, had an average fold change of 0.9 in the 1–5 mM range, but this increased to 1.7 in the 10–100 mM range. Similarly, ammonium formate, relative to formic acid, showed an average fold change of 1.1 in the lower concentration range, which increased to 2.2 in the higher concentration range. These fold changes were further supported by Figure S43a–d, which, as previously mentioned, represents the summation of peak areas for all analytes using additive concentration ranges of 1–100 mM, 2.5–100 mM, 5–100 mM, and 10–100 mM. The figure also shows that the buffer salts outperform their corresponding acids. However, there are exceptions, such as in the case of clindamycin, where acetic acid performs the best. This highlights the role of the compound’s chemical nature in influencing the signal intensity. Nevertheless, at higher additive concentrations, the signal intensity appears to be influenced by both the solvation enthalpy of the proton source and the surface tension of the ammonium salts. Regarding the solvation enthalpy, ions with more positive (less negative) solvation energies favor the droplet surface due to partial solvation, while those with more negative solvation energies prefer the interior of the droplet for complete solvation [16,17]. In the context of this study, the solvation enthalpy of NH_4_^+^ is −329 kJ/mol, while that of H_3_O^+^ is significantly more negative at −1150 ± 0.9 kJ/mol [18]. As a result, NH_4_^+^, with a much more positive solvation energy compared to H_3_O^+^, becomes more enriched on the droplet surface, leading to more efficient desorption and ultimately facilitating the ionization of compounds in the gas phase or on the droplet surface [19,20,21,22]. Furthermore, a study by Creydt and Fischer [12] on plant metabolites demonstrated that ammonium formate and ammonium acetate outperformed their acidic counterparts. They hypothesized that this enhancement was due to the ability of ammonium salts to reduce surface tension.

The second comparison will involve maintaining the same proton source (NH_4_^+^) while examining the influence of different conjugate base species—hydroxide, bicarbonate, formate, and acetate. Figure 2 demonstrates that ammonium hydroxide generally surpasses the other ammonium-based additives in performance. This was again determined by comparing the fold changes across the entire concentration range of ammonium hydroxide with those of ammonium bicarbonate, ammonium acetate, and ammonium formate, which were 1.3, 3.7, and 2.6, respectively (see Supplementary Information—Excel spreadsheet). This result was further supported by Figure S43, which shows that ammonium hydroxide generally outperforms the other ammonium-based additives. However, in certain cases, ammonium hydroxide performs comparably to ammonium bicarbonate, and for sulfamethoxazole, ammonium bicarbonate proves to be the more effective additive. The performance of ammonium hydroxide may be attributed to the higher (more negative) solvation enthalpy of the hydroxide anion (−1622.7 kJ/mol) compared to other anions, formate (−1535 kJ/mol), acetate (−1528 kJ/mol), and bicarbonate (−1487 kJ/mol). This hypothesis is based on findings from McIndoe and colleagues, who investigated the signal intensity of intrinsic cations in the presence of solvated anions (counterions) in polar and non-polar solvents [9,10]. McIndoe and colleagues’ first study [9] revealed that chloride aggregates of the 1-butyl-3-methylimidazolium cation ([(BMIM)_2_ + Cl]⁺) produced a stronger ESI-MS response than their bistriflimide counterparts ([(BMIM)_2_ + NTf_2_]⁺) in dichloromethane. This effect was attributed to chloride’s lower solvation in dichloromethane. A subsequent study by McIndoe [10] reinforced this trend in polar solvents like acetonitrile. For example, the tetraethylammonium cation exhibited a higher signal intensity in the presence of chloride anions compared to bistriflimide anions, as chloride ions experienced stronger solvation in acetonitrile. Applying these findings to our study, we observe a clear analogy with the hydroxide anion, which is more solvated than the other anions in the aqueous mobile phase, leading to increased signal intensity. While the exact mechanism of charge neutralization remains uncertain, the solvated anions (i.e., the hydroxide anion) are more likely to reside in the droplet’s interior, whereas less solvated anions (i.e., acetate and formate) can migrate to the surface, causing charge neutralization of the positively charged cations.

Another plausible explanation for ammonium hydroxide’s effectiveness as an additive, consistently outperforming ammonium formate and acetate, is its lower conductivity (except at 1 mM)—a result supported by the findings of Huber and Krajete [23], Cole and Harrata [24], and Mirza and Chait, although not explicitly stated in their work [3]. Furthermore, hydroxide is more effective than ammonium acetate and formate (except for 12-hydroxynevirapine) at removing MS signal-deteriorating metal–analyte complexes, though still less effective than bicarbonate (which will be discussed shortly; see Table S3). These factors collectively make NH_4_OH a very efficient additive in positive ion mode. On the other hand, ammonium bicarbonate, which has conductivity similar to that of ammonium acetate and formate (see Table S2), has been shown to be highly effective at preventing the formation of metal adducts—specifically [M + Na]⁺ and [M + K]⁺. This effect has been demonstrated in studies of DNA base pairs [4,5] and DNA adducts [6,7,8], ultimately leading to an increased [M + H]⁺ ion current. This study also examined this occurrence by directly infusing the molecules into the mass spectrometer in full-scan mode at a 5 mM additive concentration and measuring the ratio of [M + H]^+^/[M + Na]^+^, which is summarized in Table S3. Firstly, trimethoprim was the only compound that did not form [M + Na]^+^ adducts, due to the absence of a carbonyl or carboxyl functional group [22]. This aligns with DNA studies, in which the bicarbonate anion is most effective at reducing—and at times even eliminating—the formation of [M + Na]^+^ adducts, ultimately leading to a stronger [M + H]^+^ ion signal [6]. Also, Yin and colleagues [6] proposed that in the case of ammonium bicarbonate, HCO_3_ can convert into carbonic acid (H_2_CO_3_), which subsequently breaks down into carbon dioxide and water via thermal degradation, a mechanism that may facilitate protonation.

The last comparison, although not particularly significant, was between acetic acid and formic acid (which produce the same proton source), with acetic acid outperforming formic acid by an average fold change of 1.2 across the entire concentration range (see Supplementary Information—Excel spreadsheet). This finding is further corroborated by Figure S43, which illustrates that acetic acid generally yielded greater peak areas, although there are exceptions, such as trimethoprim (see Figure 2). Since acetate and formate have very similar solvation enthalpies, a possible explanation for the consistently higher signal intensities of acetic acid across the concentration range may be its lower surface tension, which could reduce ion evaporation [25,26]. Specifically, acetic acid has a surface tension of 27.1 mN/m, compared to 37.13 mN/m for formic acid. Furthermore, the study by Huber and Krajete [23] supports our findings through their analysis of nucleic acids in negative ionization mode, using acetic and formic acid as ion-pair reagents. They suggested that acids with lower conductivity would have the least suppressive effect on ionization, a conclusion that aligns with our conductivity measurements (see Table S2). This underscores the idea that sufficient conductivity is necessary, and that both excessively high and low conductivity can negatively impact signal intensity [24,26].

**Figure 3:**
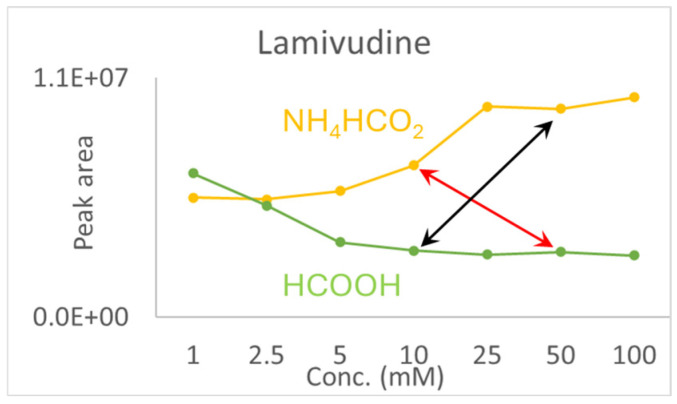
The graph of lamivudine shows that the effective concentration of the additive has no effect on the overall result.

Finally, to address the issue of effective additive concentration at the time of detection—since the additive was only present in the aqueous phase—it is important to note that, as discussed in this manuscript, notable fold changes were only observed at concentrations above 5 mM. Using lamivudine as an example, when comparing ammonium formate to formic acid, we observed longer retention times under buffer conditions for lamivudine (but also for trimethoprim and clindamycin). This suggests that the acid concentration was higher than that of the buffer concentration at the time of detection. This trend is illustrated by the red line in Figure 3 below, which shows a higher signal intensity in the presence of the buffer, at lower additive concentrations. Hypothetically, if lamivudine had shown a longer retention time under acidic conditions, the effective concentration of the buffer at the time of detection would have been higher (represented by the black line). Yet, the signal intensity remains higher with the buffer, reinforcing the conclusion that the effective additive concentration at the time of detection did not influence the overall outcome. The same argument can be applied to the remaining compounds.

## 3. Materials and Methods

### 3.1. Chemicals

Lamivudine (3TC), emtricitabine (FTC), 12-hydroxynevirapine (NVPM), nevirapine (NVP), ritonavir (RTV), trimethoprim (TMP), clindamycin (CD), and sulfamethoxazole (SMX) were obtained from ClearSynth (Mumbai, India). Ultra-purity-grade acetonitrile (ACN), methanol (MeOH), and water were purchased from Romil (Waters™ (Microsep), Johannesburg, Gauteng, South Africa). Formic acid and ammonium bicarbonate were obtained from Merck (Darmstadt, Hessen, Germany). Ammonium acetate, ammonium formate, acetic acid, and ammonium hydroxide were acquired from Sigma-Aldrich (St. Louis, MO, USA).

### 3.2. Preparation of Working Solution

A working solution containing all analytes was prepared in 20% aqueous methanol at equimolar concentrations of 200 ng/mL.

### 3.3. Electrical Conductivity (EC) Measurements

A Hanna pH/EC meter (Hanna Instruments, Inc., Smithfield, RI, USA) was used to measure EC values at additive concentrations of 1, 10, and 100 mM. Samples were placed in a water bath maintained at 25 °C and allowed to equilibrate for 15 min. The EC meter was calibrated with a 1413 µS/cm EC buffer. After each measurement, the probe was rinsed with ultrapure water to prevent cross-contamination between samples

### 3.4. Liquid Chromatography Mass Spectrometry Analysis

The analysis was carried out using an ABSciex 6500+ Qtrap mass spectrometer, connected to a Shimadzu LC-40D pump, DGU-405 degasser, Sil-40C autosampler, and CTO-40C column oven. The mass spectrometer and liquid chromatograph were both acquired from Promolab (Pty) Ltd. (Cape Town, South Africa), SEPARATIONS. Both the mass spectrometer and liquid chromatograph were controlled by Analyst^®^ Software version 1.7. Separations were performed using a Kinetex^®^ EVO C18 (1.7 μm, 50 × 2.1 mm) column kept at 30 °C. Mobile phase A consisted of aqueous solutions containing the additives investigated in this study at concentrations of 1, 2.5, 5, 10, 25, 50, and 100 mM while mobile phase B was acetonitrile (ACN). It is important to note that when switching between mobile phase additives, the liquid chromatograph was thoroughly rinsed with isopropanol, followed by ultrapure water, to ensure the complete removal of any residual additive that could potentially influence the results. The solvent gradient began at 95% A and decreased to 5% A over 6 min, followed by a 1.5-min re-equilibration, resulting in a total run time of 7.5 min. The flow rate was 0.4 mL/min, and the injection volume was 1 μL. The 6500+ QTrap mass spectrometer operating in positive electrospray ionization mode was used as a detector by applying multiple reaction monitoring (MRM). The ESI parameters were configured as follows: collisional gas was set to high; curtain gas at 45 psi; CAD at low; ion source gases 1 and 2 both at 40 psi; source temperature at 400 °C; and ion spray voltage at 5.5 kV. For each analyte, two MRM transitions were monitored. Compound-specific settings—including declustering potential (DP), collisional energy (CE), entrance potential (EP), and collision cell exit potential (CXP)—were individually optimized by direct injection under continuous flow conditions. The MRM transition along with dwell times, DP, EP, CE, and CXP values are provided in Table S1.

### 3.5. Data Handling

Excel version 2308 (Microsoft Corporation, Redmond, WA, USA) was used to construct the graphs of concentration vs. peak areas. Peak area integration was performed using Analyst^®^ software version 1.7.

## 4. Conclusions

This study highlights the influence of solvation enthalpy, surface tension, and conductivity on positive ionization efficiency in electrospray ionization mass spectrometry (ESI-MS) using common mobile phase additives. In essence, proton sources that are less solvated, and highly solvated anionic species (i.e., conjugated base species of the proton sources), tend to produce higher signal intensities in positive ionization mode. Additionally, additives that lower surface tension and do not generate excessively high conductivity are more favorable for ESI-MS. The results demonstrate that ammonium-based additives, particularly ammonium hydroxide and bicarbonate, are highly effective in positive ionization mode. Notably, while formic acid is the least effective additive, it remains widely used among mass spectrometry practitioners. To further explore the influence of counterions on ionization efficiency in both positive and negative ionization modes, future studies could build on the work of McIndoe and colleagues [9,10]. Such studies could employ intrinsic positive and negative ions with varying hydrophobicities while utilizing the common electrospray additives examined in this study. This approach could provide deeper insights into the role of solvation enthalpy in charge neutralization.

## Data Availability

Data available on request.

## Supplementary Materials

The following supporting information can be downloaded at: https://www.mdpi.com/article/10.3390/molecules30091885/s1, Table S1: Mass spectrometric parameters, Table S2: Conductivity measurements (μS/cm) at additive concentrations of 1, 10, and 100 mM, Table S3: The ratio of [M + H]^+^/[M + Na]^+^ following direct infusion into the mass spectrometer, Chromatograms representing all additives at concentrations from 1 to 100 mM are presented for acetic acid (Figures S1–S7), formic acid (Figures S8–S14), ammonium acetate (Figures S15–S21), ammonium formate (Figures S22–S28), ammonium hydroxide (Figures S29–S35), and ammonium bicarbonate (Figures S36–S42). The fold-changes are added as an additional Excel spreadsheet, named AnalyteFoldChanges. Figure S43a–d: The summation of peak areas for all analytes using additive concentration ranges of 1–100 mM, 2.5–100 mM, 5–100 mM, and 10–100 mM. Excel was used to con-struct the graphs of concentration vs. peak areas.
