# Supplementary material for: The Effects of Solvation Enthalpy, Surface Tension, and Conductivity of Common Additives on Positive Electrospray Ionization in Selected Pharmaceuticals
Source: Molecules. 2025 Apr 23;30(9):1885. doi: 10.3390/molecules30091885 (PMC12073616; doi:10.3390/molecules30091885)
Supplement: Supplementary file 1 [file molecules-30-01885-s001.zip › molecules-3538236-3rd revised - supplementary.pdf]

Supplementary

The effects of solvation enthalpy, surface tension, and conductivity of common additives on positive electrospray ionization in selected pharmaceuticals

Pieter Venter

Table S1: Mass spectrometry parameters.

Analyte	Q1 Mass	Q2 Mass	Dwell time	DP (volts)	EP (Volts)	CE (Volts)	CXP (Volts)
**Emtricitabine quantifier**	248.063	130.1	20	16	10	15	14
**Emtricitabine qualifier**	248.063	112.9	20	16	10	51	12
**Lamivudine quantifier**	230.036	112	20	11	10	17	12
**Lamivudine qualifier**	230.036	95	20	11	10	53	10
**Sulfamethoxazole quantifier**	254.049	156	20	51	10	21	18
**Sulfamethoxazole qualifier**	254.049	108	20	51	10	31	12
**Nevirapine quantifier**	267.076	226.1	20	91	10	35	14
**Nevirapine qualifier**	267.076	227.1	20	91	10	41	12
**12-Hydroxynevirapine quantifier**	283.06	265.1	20	81	10	27	20
**12-Hydroxynevirapine qualifier**	283.06	223.1	20	81	10	41	14
**Trimethoprim quantifier**	291.079	230.2	20	111	10	31	12
**Trimethoprim qualifier**	291.079	261.1	20	111	10	33	14
**Clindamycin quantifier**	425.127	126.1	20	56	10	33	6
**Clindamycin qualifier**	425.127	377.1	20	56	10	27	26
**Ritonavir quantifier**	721.226	296	20	71	10	25	22
**Ritonavir qualifier**	721.226	268.1	20	71	10	37	22

Table S2: Conductivity measurements (µS/cm) at additive concentrations of 1, 10, and 100 mM.

	1 mM	10 mM	100 mM
**HCOOH**	148	531	1723
**CH_3COOH**	64	165	502
**NH_4HCO_2**	14	120	1107
**$NH_4CH_3CO_2$**	15	118	1002
**NH_4HCO_3**	15	117	1012
**NH_4OH**	38	86	264

Table S3: The ratio of $[M+H]^+/[M+Na]^+$ following direct infusion into the mass spectrometer.

Analyte	Ammonium formate	Formic acid	Ammonium acetate	Acetic acid	Ammonium hydroxide	Ammonium bicarbonate
**Lamivudine**	0.7	2	0.5	1.5	5.0	38.0
**Emtricitabine**	0.7	0.8	0.6	0.2	2.6	18.0
**Sulfamethoxazole**	1.2	0.6	0.8	0.4	5.9	21.0
**Nevirapine**	2.6	No adduct	16.0	8.7	No Adduct	No Adduct
**12 Hydroxynevirapine**	14.0	No adduct	9.0	3.0	3.8	No Adduct
**Trimethoprim**	No Adduct	No adduct	No Adduct	No adduct	No Adduct	No Adduct
**Clindamycin**	7.0	13	3.0	11.8	11.6	19.0
**Ritonavir**	2.2	1.2	2.0	0.4	4.7	26.0

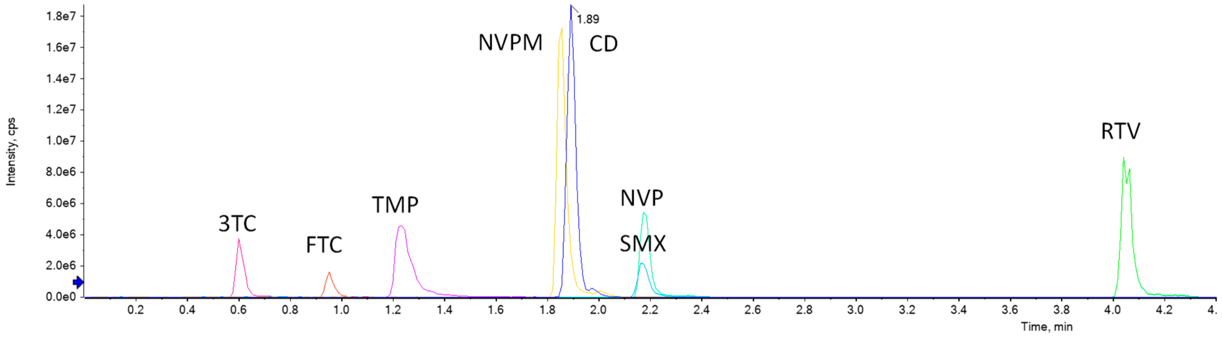

Figure S1: MRM chromatogram illustrating analyte peaks obtained using 1 mM acetic acid as mobile phase additive. Refer to Figure 1 and section 3.1 for the abbreviations used in the peak notations.

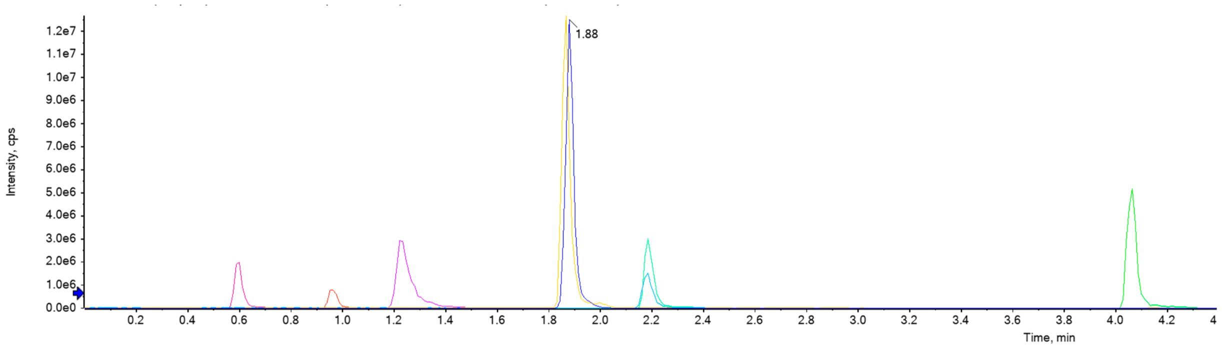

Figure S2: MRM chromatogram illustrating analyte peaks obtained using 2.5 mM acetic acid as mobile phase additive.

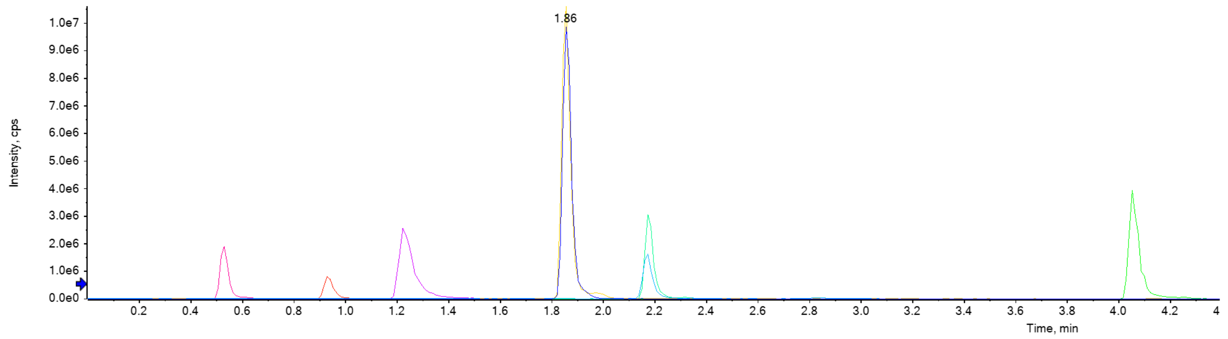

Figure S3: MRM chromatogram illustrating analyte peaks obtained using 5 mM acetic acid as mobile phase additive.

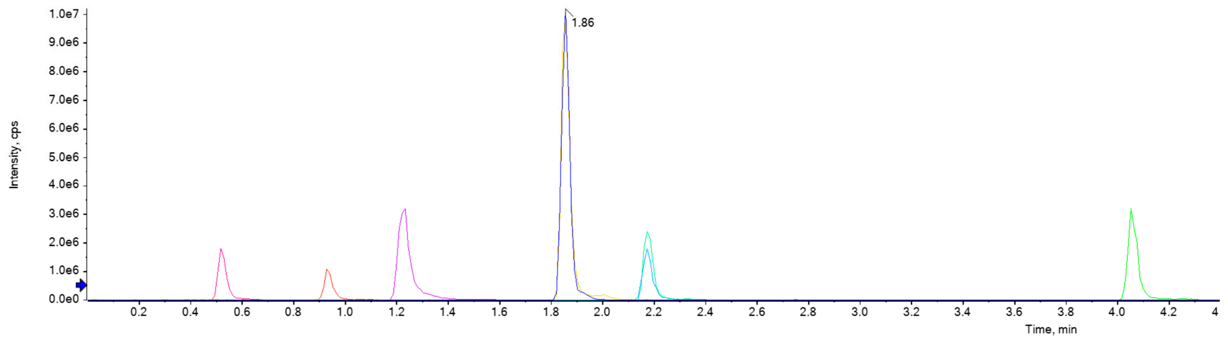

Figure S4: MRM chromatogram illustrating analyte peaks obtained using 10 mM acetic acid as mobile phase additive.

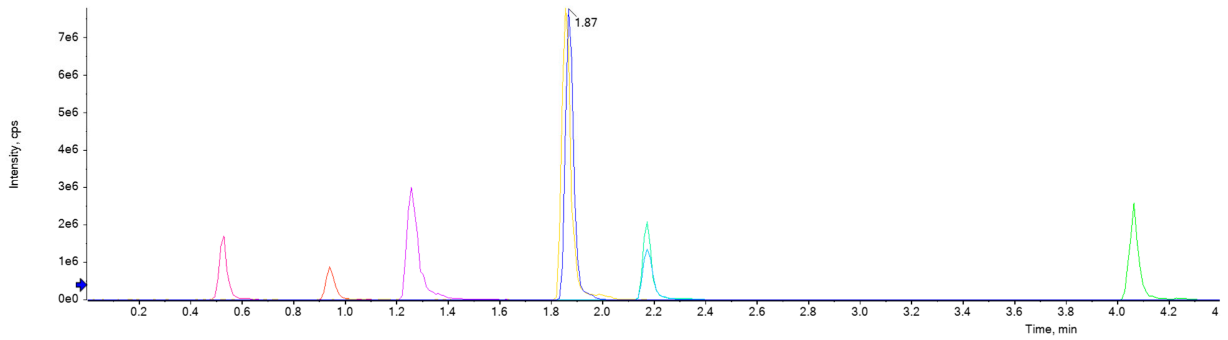

Figure S5: MRM chromatogram illustrating analyte peaks obtained using 25 mM acetic acid as mobile phase additive.

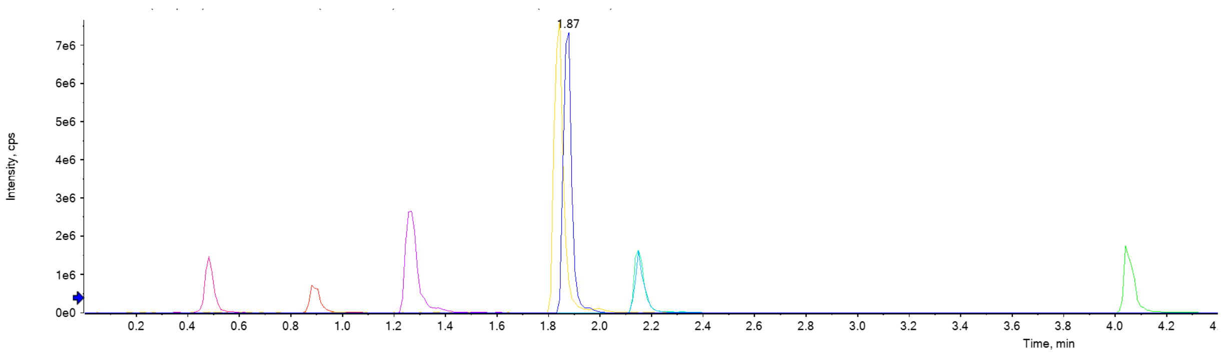

Figure S6: MRM chromatogram illustrating analyte peaks obtained using 50 mM acetic acid as mobile phase additive.

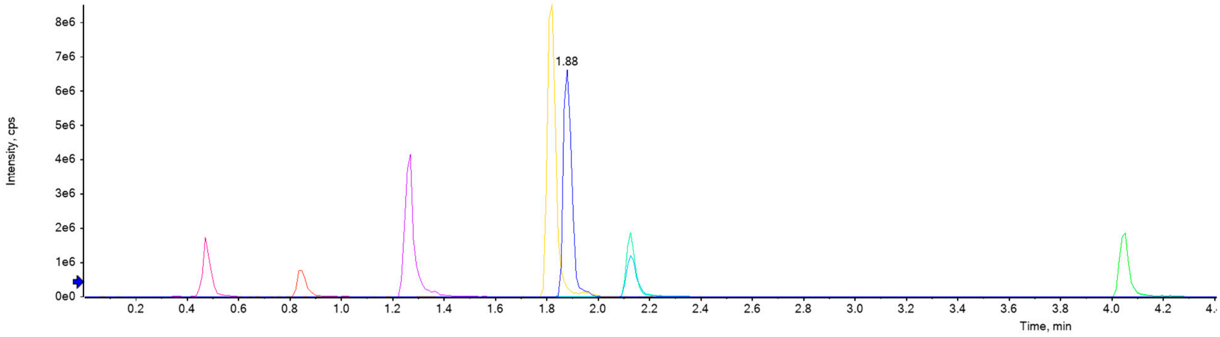

Figure S7: MRM chromatogram illustrating analyte peaks obtained using 100 mM acetic acid as mobile phase additive.

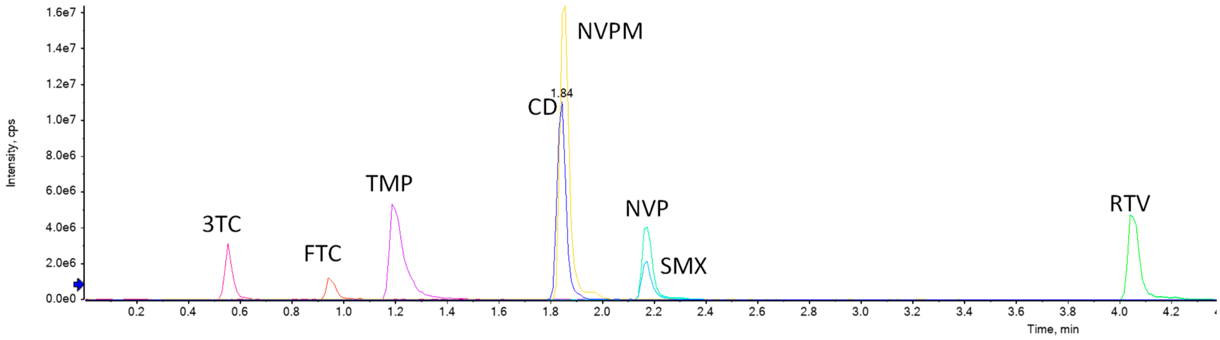

Figure S8: MRM chromatogram illustrating analyte peaks obtained using 1 mM formic acid as mobile phase additive.

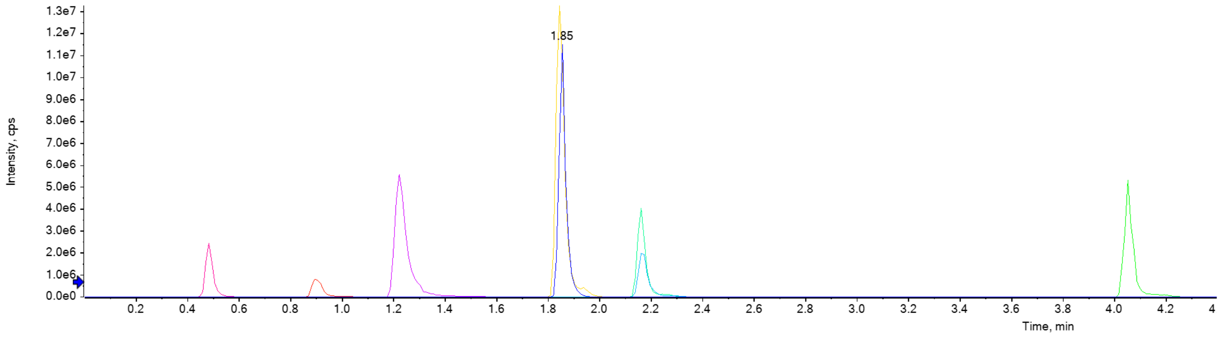

Figure S9: MRM chromatogram illustrating analyte peaks obtained using 2.5 mM formic acid as mobile phase additive.

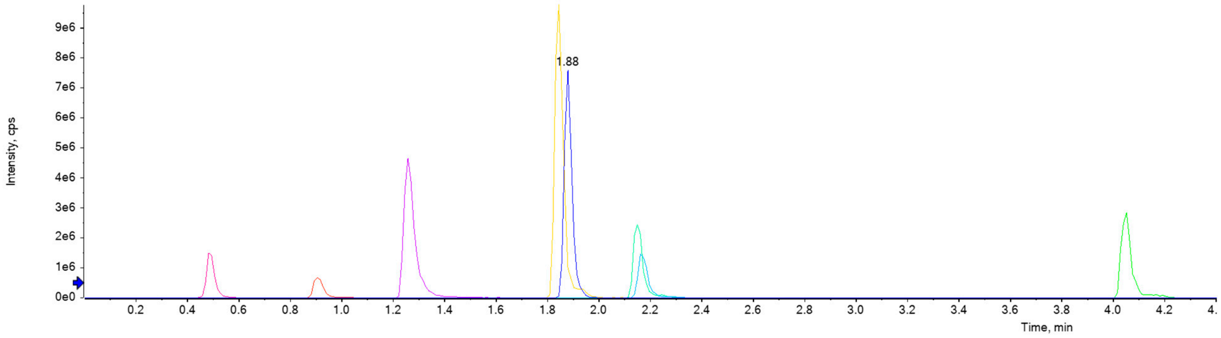

Figure S10: MRM chromatogram illustrating analyte peaks obtained using 5 mM formic acid as mobile phase additive.

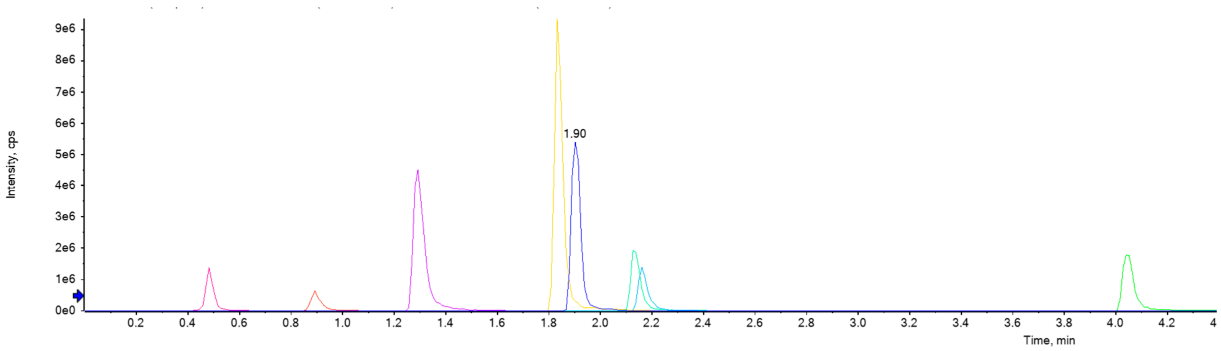

Figure S11: MRM chromatogram illustrating analyte peaks obtained using 10 mM formic acid as mobile phase additive.

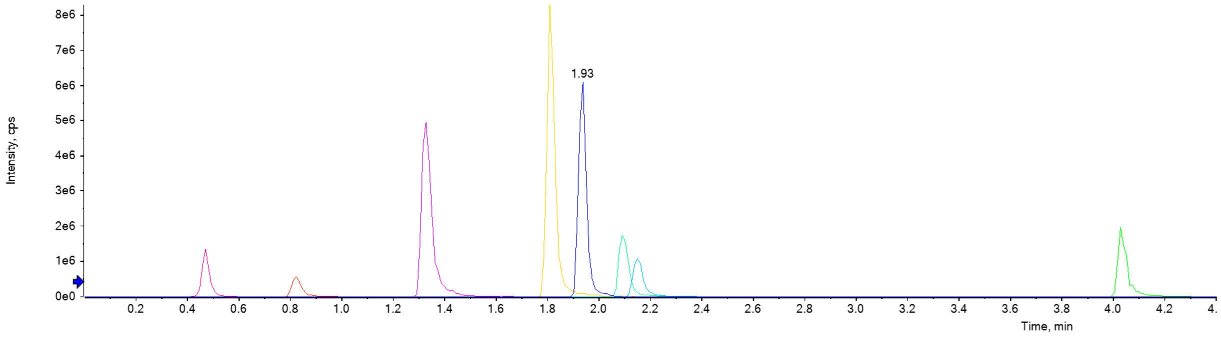

Figure S12: MRM chromatogram illustrating analyte peaks obtained using 25 mM formic acid as mobile phase additive.

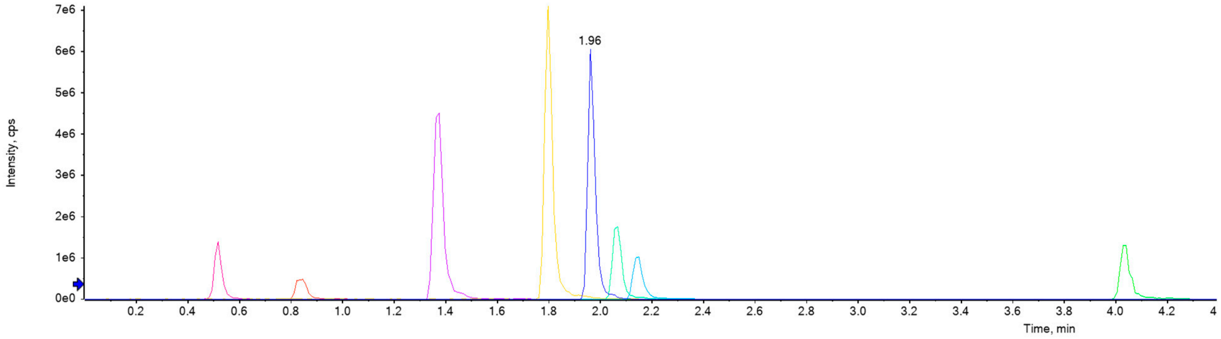

Figure S13: MRM chromatogram illustrating analyte peaks obtained using 50 mM formic acid as mobile phase additive.

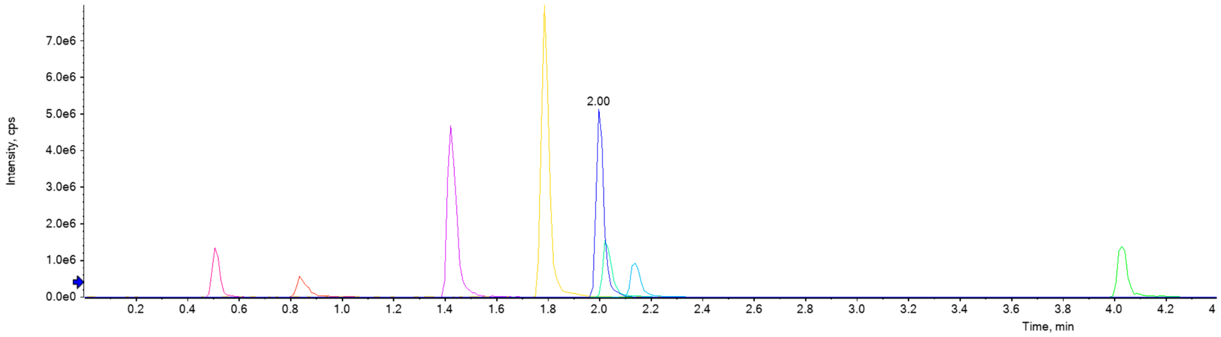

Figure S14: MRM chromatogram illustrating analyte peaks obtained using 100 mM formic acid as mobile phase additive.

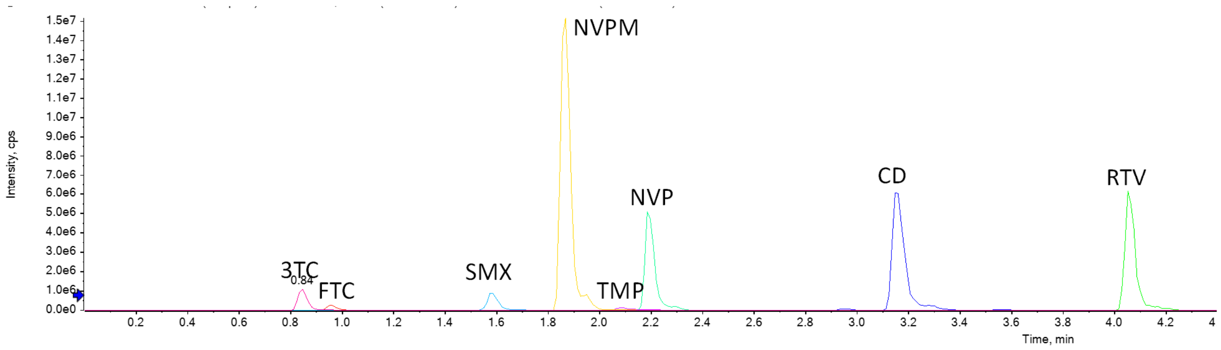

Figure S15: MRM chromatogram illustrating analyte peaks obtained using 1 mM ammonium acetate as mobile phase additive.

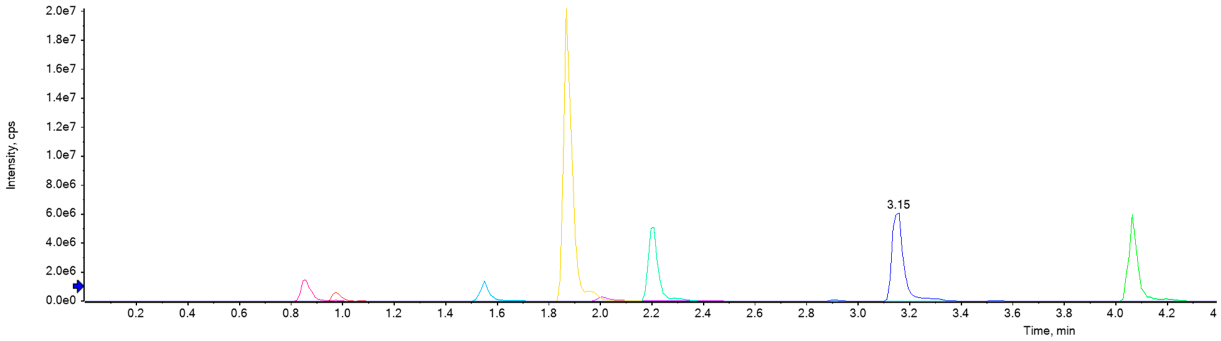

Figure S16: MRM chromatogram illustrating analyte peaks obtained using 2.5 mM ammonium acetate as mobile phase additive.

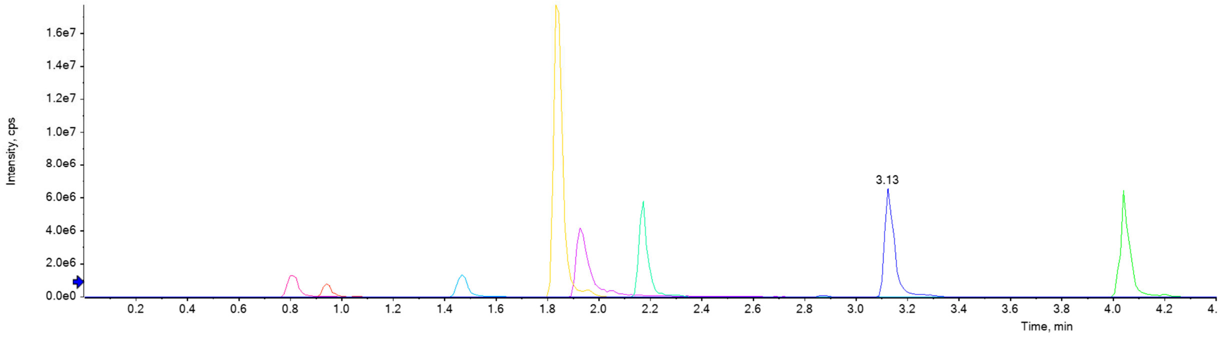

Figure S17: MRM chromatogram illustrating analyte peaks obtained using 5 mM ammonium acetate as mobile phase additive.

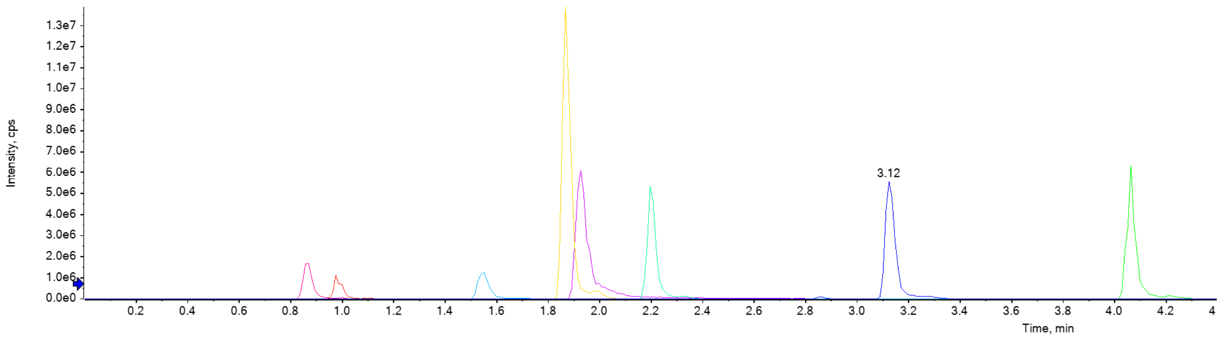

Figure S18: MRM chromatogram illustrating analyte peaks obtained using 10 mM ammonium acetate as mobile phase additive.

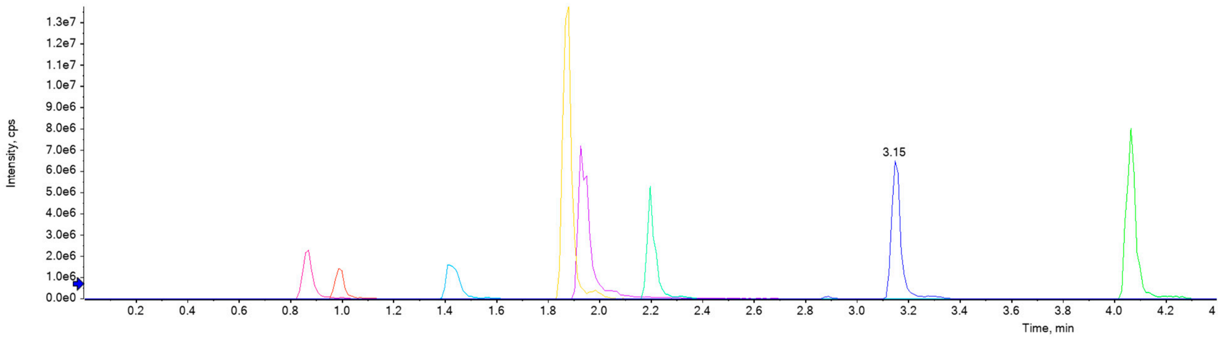

Figure S19: MRM chromatogram illustrating analyte peaks obtained using 25 mM ammonium acetate as mobile phase additive.

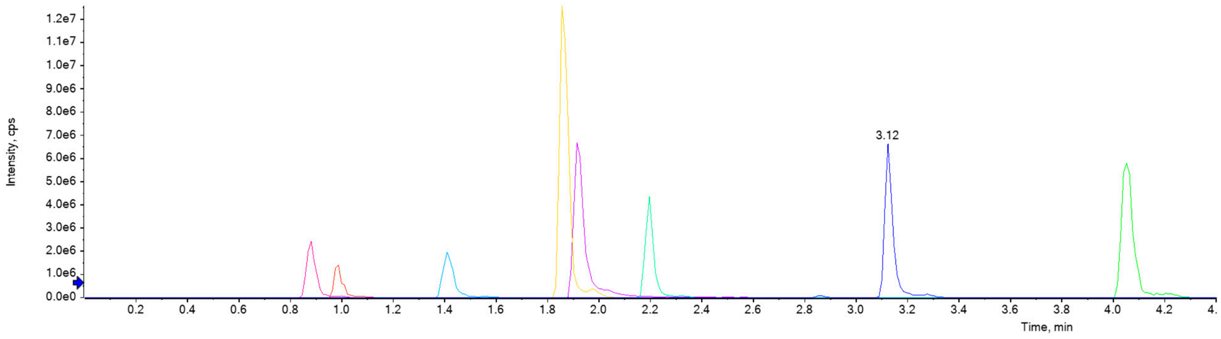

Figure S20: MRM chromatogram illustrating analyte peaks obtained using 50 mM ammonium acetate as mobile phase additive.

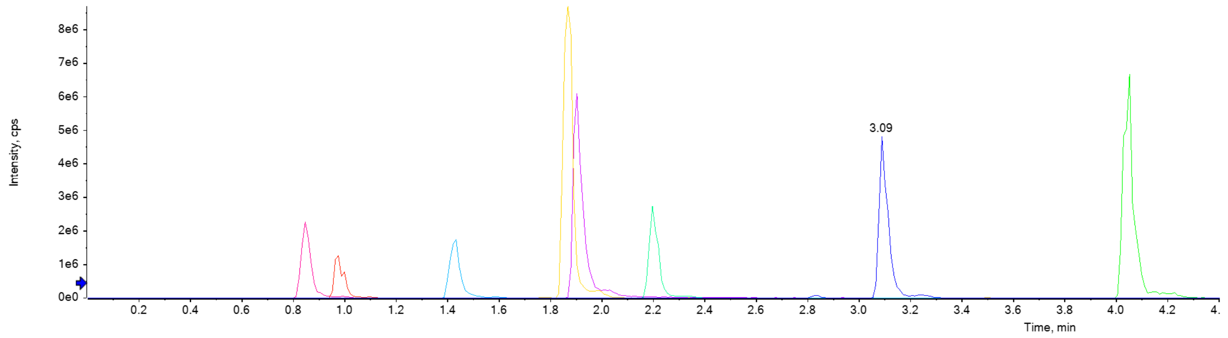

Figure S21: MRM chromatogram illustrating analyte peaks obtained using 100 mM ammonium acetate as mobile phase additive.

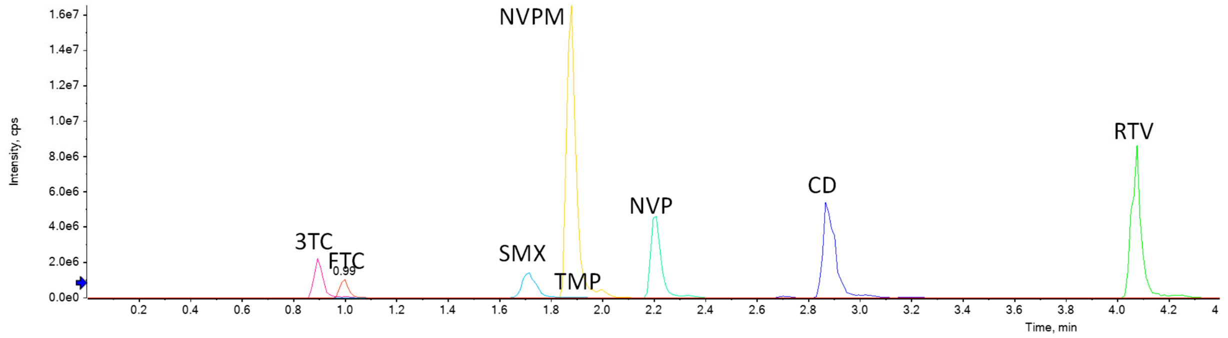

Figure S22: MRM chromatogram illustrating analyte peaks obtained using 1 mM ammonium formate as mobile phase additive.

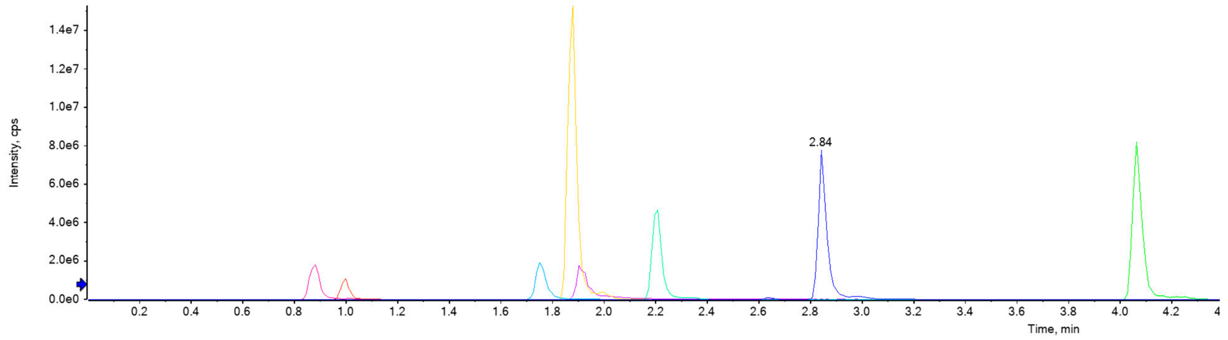

Figure S23: MRM chromatogram illustrating analyte peaks obtained using 2.5 mM ammonium formate as mobile phase additive.

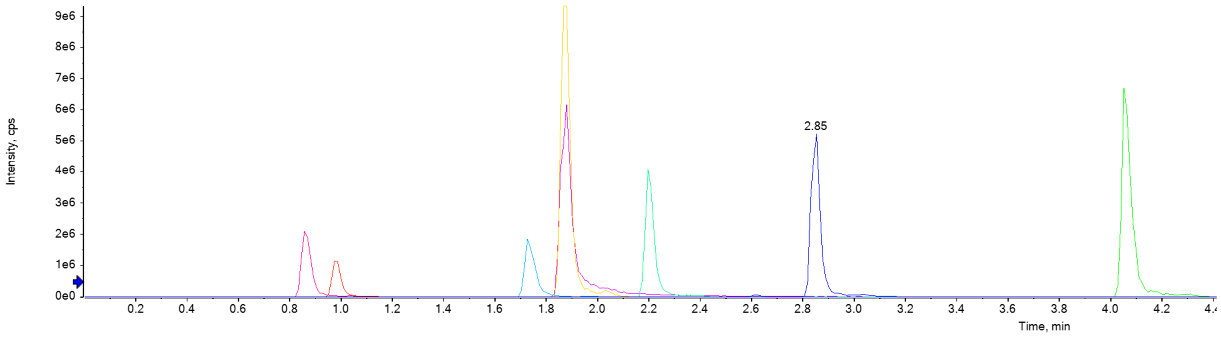

Figure S24: MRM chromatogram illustrating analyte peaks obtained using 5 mM ammonium formate as mobile phase additive.

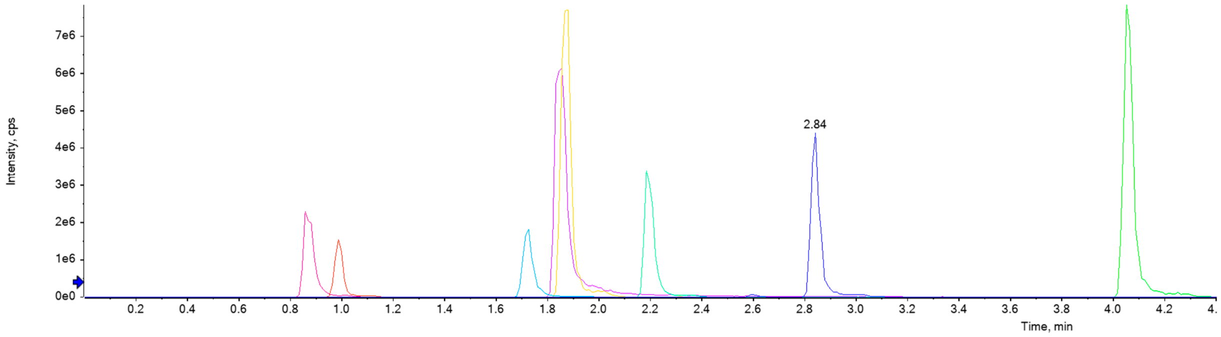

Figure S25: MRM chromatogram illustrating analyte peaks obtained using 10 mM ammonium formate as mobile phase additive.

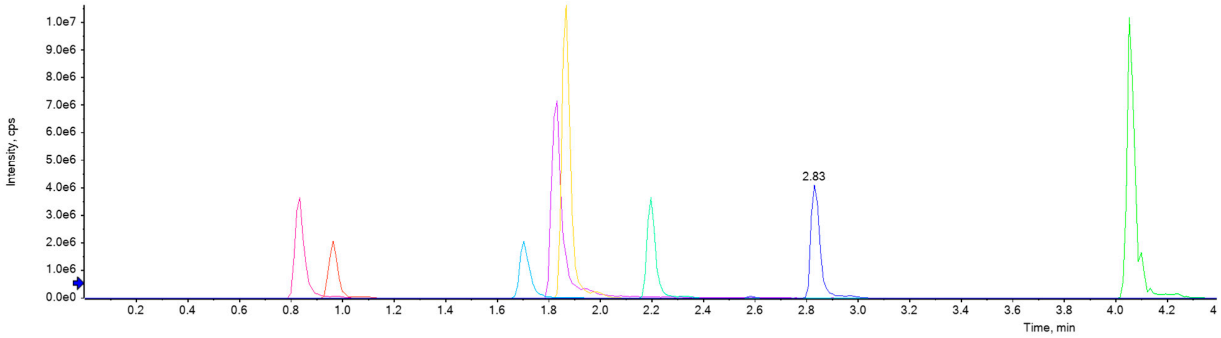

Figure S26: MRM chromatogram illustrating analyte peaks obtained using 25 mM ammonium formate as mobile phase additive.

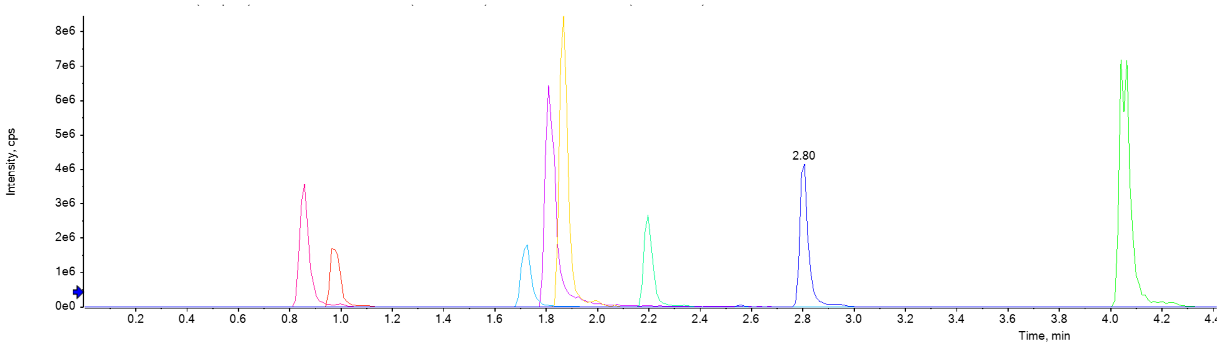

Figure S27: MRM chromatogram illustrating analyte peaks obtained using 50 mM ammonium formate as mobile phase additive.

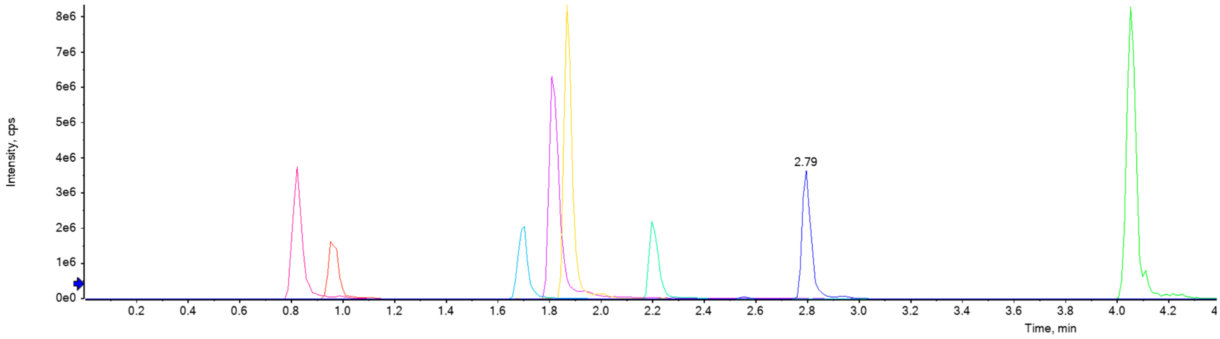

Figure S28: MRM chromatogram illustrating analyte peaks obtained using 100 mM ammonium formate as mobile phase additive.

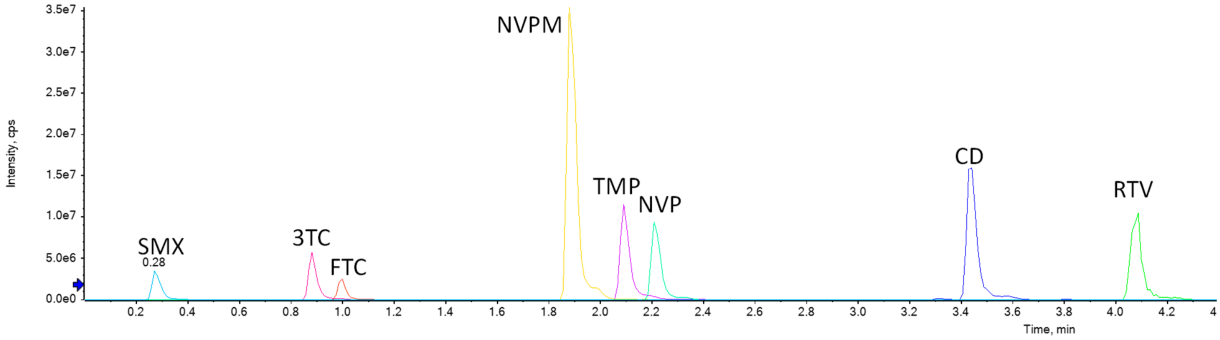

Figure S29: MRM chromatogram illustrating analyte peaks obtained using 1 mM ammonium hydroxide as mobile phase additive.

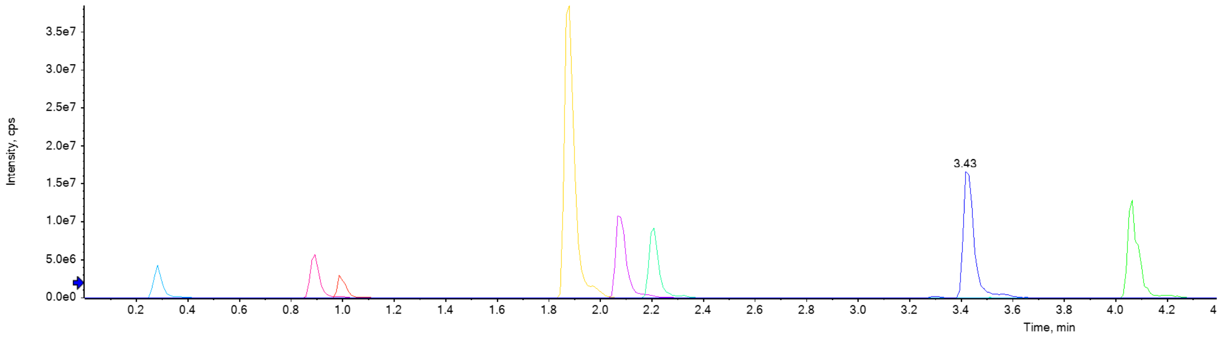

Figure S30: MRM chromatogram illustrating analyte peaks obtained using 2.5 mM ammonium hydroxide as mobile phase additive.

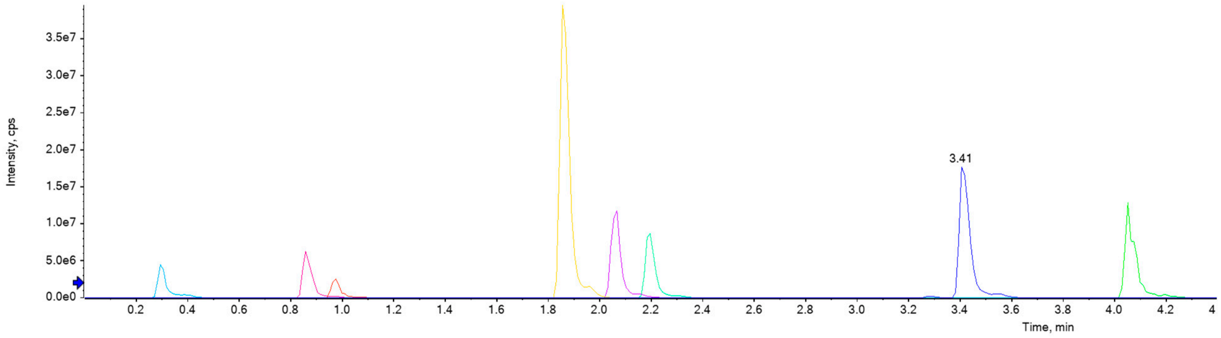

Figure S31: MRM chromatogram illustrating analyte peaks obtained using 5 mM ammonium hydroxide as mobile phase additive.

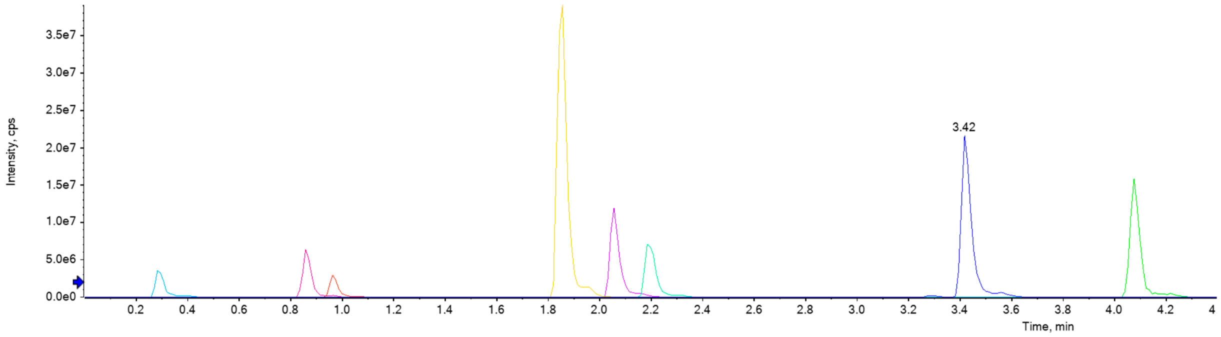

Figure S32: MRM chromatogram illustrating analyte peaks obtained using 10 mM ammonium hydroxide as mobile phase additive.

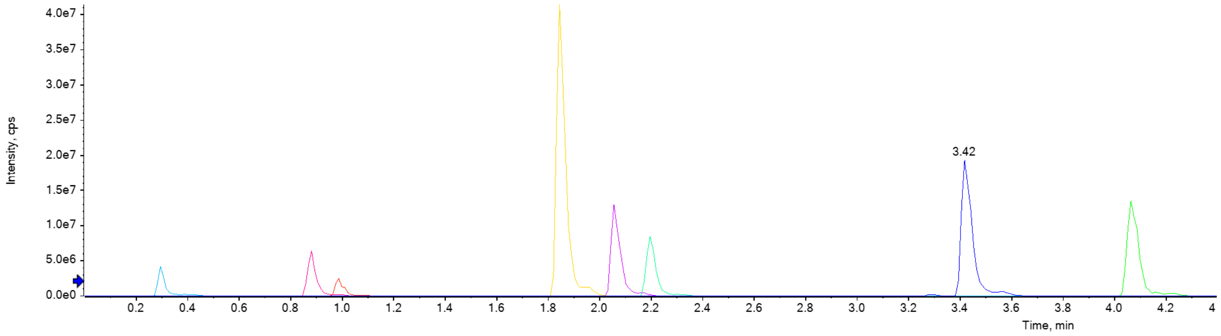

Figure S33: MRM chromatogram illustrating analyte peaks obtained using 25 mM ammonium hydroxide as mobile phase additive.

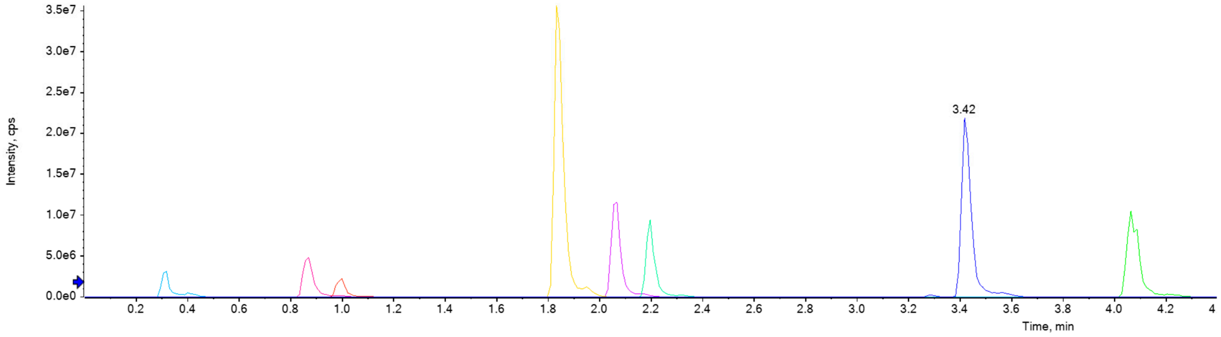

Figure S34: MRM chromatogram illustrating analyte peaks obtained using 50 mM ammonium hydroxide as mobile phase additive.

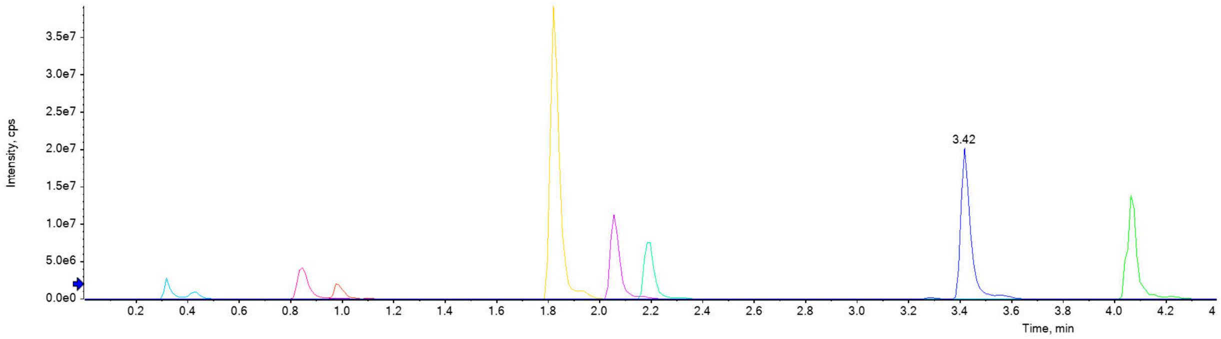

Figure S35: MRM chromatogram illustrating analyte peaks obtained using 100 mM ammonium hydroxide as mobile phase additive.

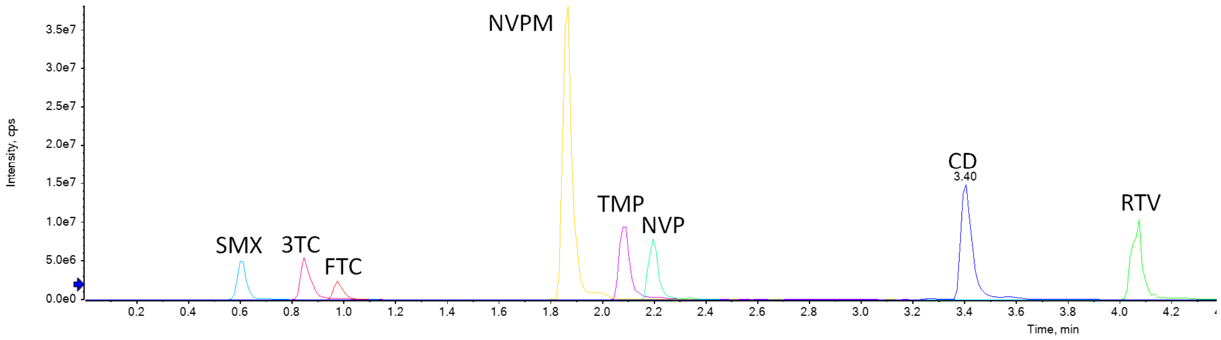

Figure S36: MRM chromatogram illustrating analyte peaks obtained using 1 mM ammonium bicarbonate as mobile phase additive.

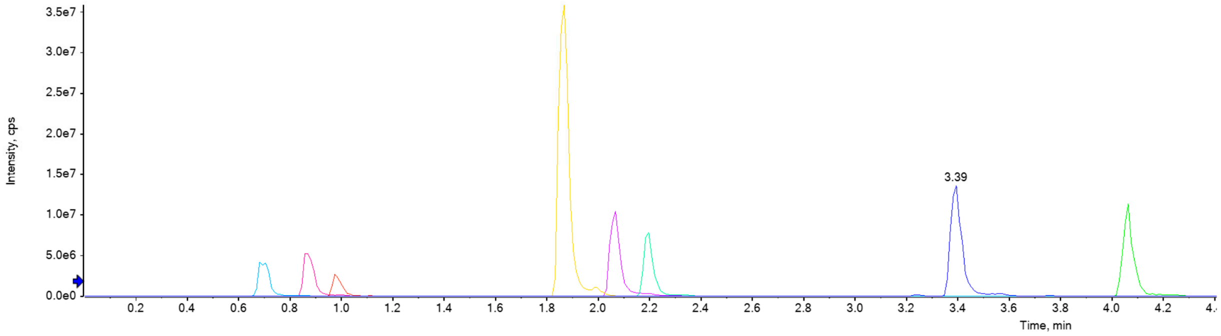

Figure S37: MRM chromatogram illustrating analyte peaks obtained using 2.5 mM ammonium bicarbonate as mobile phase additive.

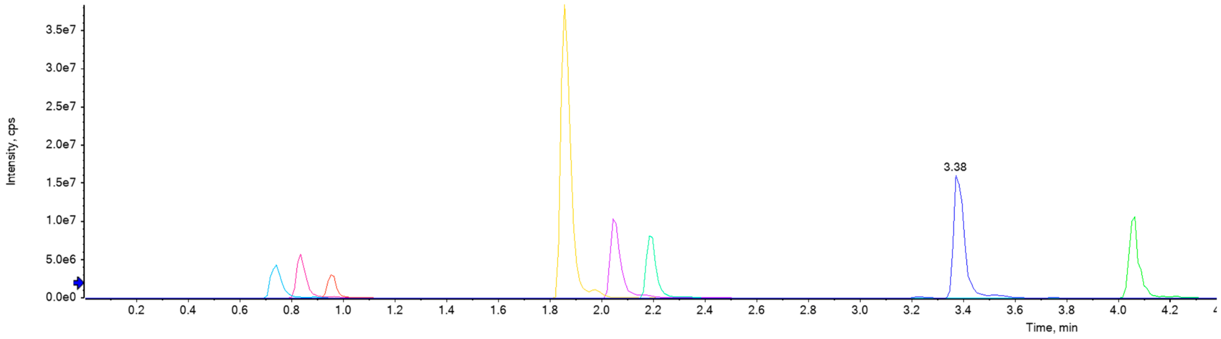

Figure S38: MRM chromatogram illustrating analyte peaks obtained using 5 mM ammonium bicarbonate as mobile phase additive.

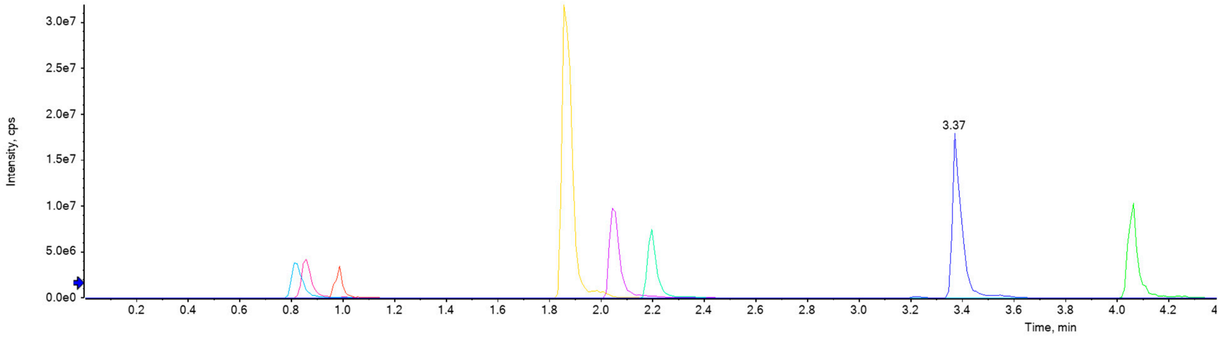

Figure S39: MRM chromatogram illustrating analyte peaks obtained using 10 mM ammonium bicarbonate as mobile phase additive.

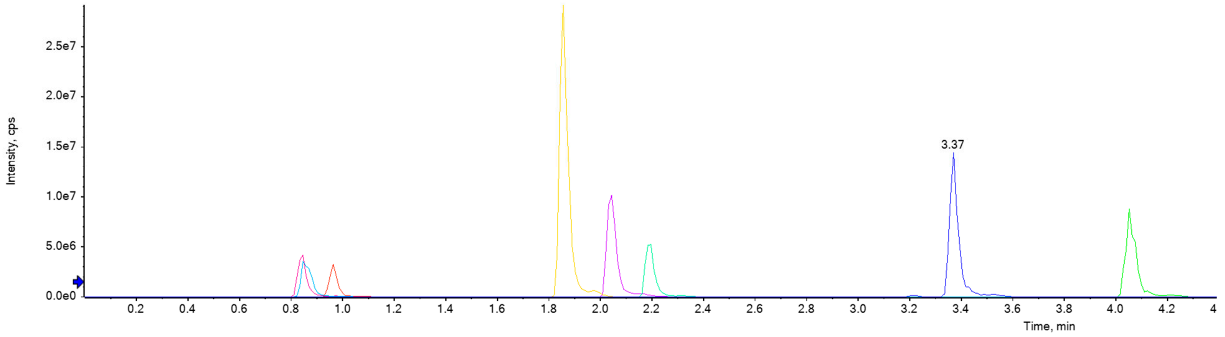

Figure S40: MRM chromatogram illustrating analyte peaks obtained using 25 mM ammonium bicarbonate as mobile phase additive.

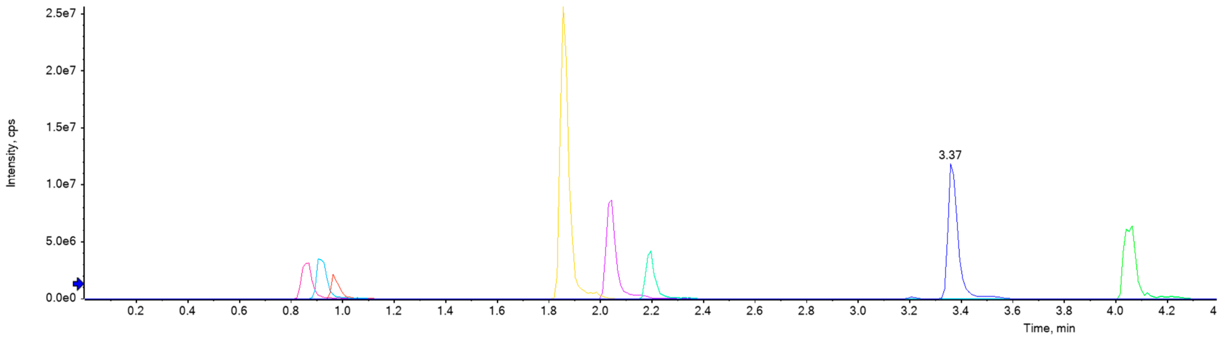

Figure S41: MRM chromatogram illustrating analyte peaks obtained using 50 mM ammonium bicarbonate as mobile phase additive.

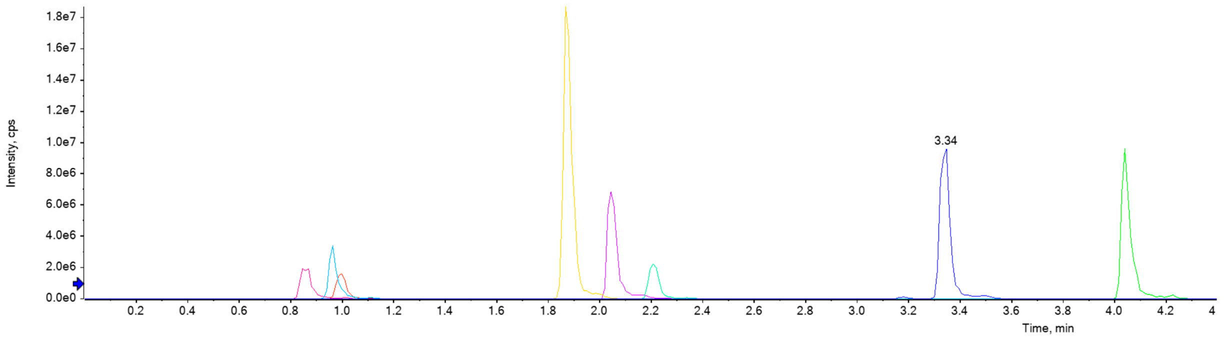

Figure S42: MRM chromatogram illustrating analyte peaks obtained using 100 mM ammonium bicarbonate as mobile phase additive.

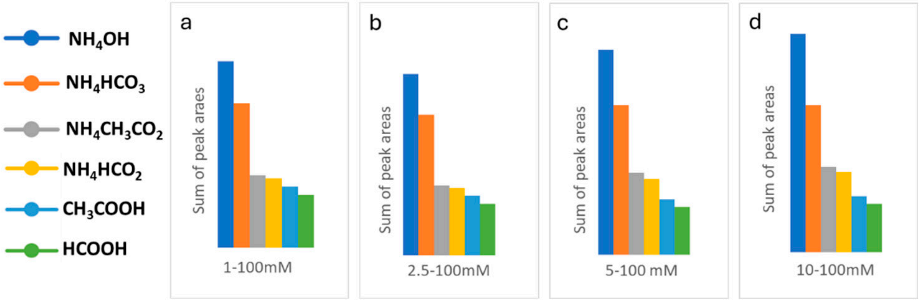

Figure S43a-d: The summation of peak areas for all analytes using additive concentration ranges of 1-100 mM, 2.5-100 mM, 5-100 mM, and 10-100 mM.
